# Integration of Functional Human Auditory Neural Circuits Based on a 3D Carbon Nanotube System

**DOI:** 10.1002/advs.202309617

**Published:** 2024-06-18

**Authors:** Yiyun Lou, Jiaoyao Ma, Yangnan Hu, Xiaoying Yao, Yaoqian Liu, Mingxuan Wu, Gaogan Jia, Yan Chen, Renjie Chai, Mingyu Xia, Wenyan Li

**Affiliations:** ^1^ ENT Institute and Otorhinolaryngology Department of Eye & ENT Hospital State Key Laboratory of Medical Neurobiology and MOE Frontiers Center for Brain Science Fudan University Shanghai 200031 China; ^2^ Institutes of Biomedical Sciences Fudan University Shanghai 200032 China; ^3^ State Key Laboratory of Digital Medical Engineering Department of Otolaryngology Head and Neck Surgery Zhongda Hospital School of Life Sciences and Technology Advanced Institute for Life and Health Jiangsu Province High‐Tech Key Laboratory for Bio‐Medical Research Southeast University Nanjing 210096 China; ^4^ Co‐Innovation Center of Neuroregeneration Nantong University Nantong 226001 China; ^5^ Obstetrics and Gynecology Hospital Fudan University Shanghai 200011 China; ^6^ NHC Key Laboratory of Hearing Medicine Fudan University Shanghai 200031 China; ^7^ The Institutes of Brain Science and the Collaborative Innovation Center for Brain Science Fudan University Shanghai 200032 China

**Keywords:** auditory cortex, organoid, spiral ganglion neuron, super‐aligned carbon nanotube sheets, synapse

## Abstract

The physiological interactions between the peripheral and central auditory systems are crucial for auditory information transmission and perception, while reliable models for auditory neural circuits are currently lacking. To address this issue, mouse and human neural pathways are generated by utilizing a carbon nanotube nanofiber system. The super‐aligned pattern of the scaffold renders the axons of the bipolar and multipolar neurons extending in a parallel direction. In addition, the electrical conductivity of the scaffold maintains the electrophysiological activity of the primary mouse auditory neurons. The mouse and human primary neurons from peripheral and central auditory units in the system are then co‐cultured and showed that the two kinds of neurons form synaptic connections. Moreover, neural progenitor cells of the cochlea and auditory cortex are derived from human embryos to generate region‐specific organoids and these organoids are assembled in the nanofiber‐combined 3D system. Using optogenetic stimulation, calcium imaging, and electrophysiological recording, it is revealed that functional synaptic connections are formed between peripheral neurons and central neurons, as evidenced by calcium spiking and postsynaptic currents. The auditory circuit model will enable the study of the auditory neural pathway and advance the search for treatment strategies for disorders of neuronal connectivity in sensorineural hearing loss.

## Graphical abstract

The lack of therapeutic avenues for auditory neural pathway disorders is primarily attributed to inadequate research models. This study pioneers a novel auditory circuit model using super‐aligned carbon nanotubes. The peripheral‐central neural organoids with functional synaptic transmission facilitate the exploration of the auditory neural pathway and propel the quest for strategies for neuronal connectivity disorders in sensorineural hearing loss.

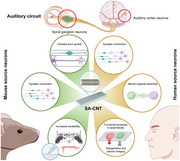

## 1 Introduction

Hearing loss is the sensory defect disease with the highest incidence rate in humans, and sensorineural hearing loss (SNHL) caused by loss or dysfunction of auditory hair cells (HCs) or neurons accounts for the majority.^[^ ^1^ ^]^ Due to the high incidence rate and intractability, SNHL affects the daily lives of hundreds of millions of people worldwide and costs hundreds of billions of dollars in public health care globally. Currently, there are very limited measures for the treatment of sensorineural cochlea, including cochlear implantation, as well as promising gene correction and regeneration therapy. For patients with profound hearing loss or complete deafness, cochlear implantation (CI) serves as the primary therapeutic method.^[^ ^2^ ^]^ CI directly stimulates spiral ganglion neurons (SGNs), which can partially substitute for the function of HCs.^[^ ^3^ ^]^ However, individuals afflicted with severe malformations of the cochlea or dysfunction of the auditory neural pathway cannot be served with the CI. The lack of effective treatment for auditory neural pathway disorders is mainly due to the lack of effective research models to develop therapeutic strategies.

The perception of acoustic information relies on the conversion of physical stimulus into electrical signals by mechanosensory HCs, transmitted by SGNs and passed through the cochlear nucleus (CN) and lateral lemniscus complex, the inferior colliculus in the midbrain, the medial geniculate nucleus of the thalamus, and received by auditory cortical neurons.^[^ ^4^ ^]^ Dysfunction in any part of the auditory pathway can lead to SNHL, hence it is urgent to establish research models for the auditory pathway. Currently, the vast majority of biological research focuses on the peripheral auditory system, especially HCs. We recently established a peripheral auditory “mini‐organ” with functional synaptic connections between HCs and SGNs in a 3D organoid co‐culture system,^[^ ^5^ ^]^ which provides a promising model for studying the peripheral auditory system. Studies have also tried to establish the connection between the central cortex and CN neurons in vitro, and limited synapses were formed in the co‐culture system of human neural precursor cell line‐derived neurons from the forebrain with rat auditory brainstem slices.^[^ ^6^ ^]^ Efforts to establish the connection between SGNs and CN neurons were made, and thrombospondin‐1 (TSP1) was used to improve synapse formation between mouse neural stem cell‐derived spiral ganglion‐like neurons and mouse CN neurons.^[^ ^7^ ^]^ Similarly, TSP1 facilitated mouse embryonic stem cell (ESC)‐derived spiral ganglion neuron‐like cells to form synapses with native mouse CN neurons. However, a model for the entire auditory nerve pathway from the start point—SGNs to the termination—auditory cortex neurons (ACNs) is still lacking.

The difficulty in establishing connections for auditory neural pathways lies in the fact that SGNs project to ACNs through several levels of neural nuclei, but emerging organoid co‐culture technologies inspire the construction of auditory nerve models. Assembled human‐induced pluripotent stem (hiPS) cell‐derived cerebral cortex spheroids, hindbrain/cervical spinal cord spheroids, and skeletal muscle spheroids formed physiologically relevant connections, which resemble functional cortico‐motor pathways.^[^ ^8^ ^]^ The model of the visual pathway was successfully established, as retinotectal projections with hiPS cell‐derived retinal, thalamic, and cortical organoids were constructed in suspension culture.^[^ ^9^ ^]^ Coculture of hiPS cell‐derived retinal and brain organoids demonstrated the formation of nerve‐like structures in which retinal ganglion cell axonal projections cross the retinal organoid to populate brain organoid regions.^[^ ^10^ ^]^ These self‐assembled neural pathways in suspension systems provide platforms for studying cell interactions and axon regeneration between specific regions. As auditory nerves are organized in an orderly pattern in the cochlea and exhibit long‐distance projection toward the central cortex throughout the auditory pathway,^[^ ^11^ ^]^ the auditory neural model should persevere orderly axonal growth for studying the reconstruction of the auditory pathway. The topography of the extracellular microenvironment was proven to influence cell morphology, provide conduction guidance, and direct cell differentiation.^[^ ^12^ ^]^ Scaffolds for guiding neurite extension in the desired direction to build synapses are urgently needed for constructing auditory pathway models.

Herein, we constructed a human auditory peripheral‐central circuit on a super‐aligned carbon nanotube sheets (SA‐CNTs)‐based 3D system with long‐range functional connections. SA‐CNTs maintain the survival of primary mouse SGNs and ACNs, and the patterned structural substrates guide the oriented growth of the two types of neural fibers from both mouse and human sources. The substrate conductivity enhanced the excitability of ion channel activity in mouse SGNs, and two primary neurons formed synaptic connections under the patterned guidance. Finally, we generated human auditory region‐specific neural spheroids from primary peripheral and central neural progenitor cells, and the differentiated SGNs and ACNs formed physiologically and functionally relevant connections assembled on the SA‐CNT‐based 3D system. The study represents an important step toward reconstructing auditory projections in a dish and a novel platform for studying auditory neural pathway dysfunction in neurodegenerative diseases as well as a more physiologically relevant system for screening pharmaceutical and genetic strategies to restore auditory pathways.

## 2 Experimental Section

### 2.1 Animals

*Bhlhb5*‐cre mice were a kind gift from Dr. Lin Gan (University of Rochester, New York), and Rosa26‐tdTomato reporter mice were purchased from the Jackson Laboratory (Stock NO. 007914). The animals were bred in the Fudan University Animal Facility to acquire pups. The feeding room was maintained at a controlled temperature of 22 ± 1 °C, a humidity of 30‐70%, and a 12 h light‐dark cycle. The wild‐type mice used were the C57BL/6J strain.

### 2.2 Animal Research

Reporting of in vivo experiments (ARRIVE) guidelines were followed in the present study. All animal experiments were approved by the Institutional Animal Care and Use Committee of Fudan University (China, 202107010S).

### 2.3 Genotyping

All transgenic mice were genotyped by PCR. Extraction of genomic DNA from mouse tails and the PCR amplification system were accomplished with a One Step Mouse Genotyping Kit (Vazyme). PCR was performed using the C1000 Touch Thermal Cycler (Bio‐Rad), according to the following scheme: an initial hot start 3 min at 94 °C, followed by 35 cycles of 94 °C for 30 s for denaturing, annealing for 30 s (58 °C for *Bhlhb5*‐cre; 61 °C for tdTomato), 72 °C for 30 s for elongation, and finally 3 min at 72 °C for final extension. The genotyping primers were as follows:

*Bhlhb5*‐wild type: (F) GACAGCGACGGCCGCT, (R) GTGCACTGTTTGCAG; *Bhlhb5*‐cre: (F) GGGATTGGACTCAGAGGCGGTAGC, (R) GCCCAAATGTTGCTGGATAGT. tdTomato: wild‐type (F) AAGGGAGCTGCAGTGGAGTA, (R) CCGAAAATCTGTGGGAAGTC; mutant (F) CTGTTCCTGTACGGCATGG, (R) GGCATTAAAGCAGCGTATCC. A 1.5% agarose gel (w/v) was applied for electrophoresis. PCR genotyping primer sets generated 623 bp *Bhlhb5*‐wild‐type and 590 bp *Bhlhb5*‐cre bands; 297 bp tdTomato wild‐type bands and 196 bp tdTomato mutant bands respectively.

### 2.4 Human Tissue Isolation

The inner ear and the cortex tissue were obtained from aborted human fetuses ranging from W10 to W16 post‐conception. The donors gave informed consent for the procurement and use of the tissues in research after deciding to terminate pregnancy and before the procedure. The medical staff who provided information about the research project had no conflicts of interest. The research team was notified of the donation only afterward. The Ethics Committee of Affiliated Eye and ENT Hospital of Fudan University, China and Obstetrics and Affiliated Gynecology Hospital of Fudan University, China approved the procurement and procedures (Project ID: 2022166, 202342). All the experiments followed the guidelines of the current version of the Declaration of Helsinki (DoH). Tissues were collected and dissected in ice‐cold PBS on the same day, as soon as possible after the procedure, otherwise properly handled.

### 2.5 Preparation and Characterization of SA‐CNTs

The SA‐CNT samples were obtained from the Nanotechnology Research Center of Tsinghua University, and they were grown using the chemical vapor deposition (CVD) method. Subsequently, the surface morphology of the SA‐CNT was characterized using scanning electron microscopy (Thermo Fisher, Quattro S, USA) and atomic force microscopy (Bruker, Dimenson ICON, USA). The conductivity of the SA‐CNT sheets was tested in parallel and perpendicular directions to the carbon tube axis using a traditional two‐probe technique. Both sides of the SA‐CNT were attached to the probe of the electrical test system, and then a specified procedure was selected for the system. The water contact angles of TCPS and SA‐CNT with or without PDL modification were measured by a drop shape analyzer (KRUSS, DSA100, Germany).

### 2.6 Culture of Primary SGNs and ACNs

The auditory cortex tissue and cochleae of mice were dissected on postnatal day (P) 2–3 and collected in ice‐cold PBS (Hyclone). The targeted human tissues were obtained from aborted human fetuses ranging from post‐conception week (PCW) 14–16. The stria vascularis and the organ of Corti were carefully removed. Then, the SGNs were detached from the modiolus by fine forceps and incubated in 0.125% trypsin‐EDTA for 15 min in a 37  °C incubator (Thermo Fisher Scientific). The cortex tissue was digested for 7 min at 37 °C. Digestion was terminated by adding an equal volume of 10% FBS in DMEM/F12 medium (Thermo Fisher Scientific). Then, the SGN and cortex tissue were washed twice with DMEM and gently triturated with a 200 µL pipette. The cells were resuspended in plating medium (DMEM/F12 supplemented with 10% FBS and 100 µg mL^−1^ penicillin/streptomycin) and then passed through a 40 µm cell strainer. The prepared SA‐CNT and tissue culture polystyrene (TCPS) were sterilized with UV light for at least 8 h. After overnight incubation in poly‐D‐lysine (Thermo Fisher Scientific) at 37 °C, the substrates were then washed 3 times with PBS and dried on a clean bench. Neurons (1 × 105 cells cm^−2^) were seeded on either SA‐CNTs or TCPS and kept at 37 °C and 5% carbon dioxide (CO_2_). After 2–3 h, the medium was replaced with differentiation medium, which was based on DMEM/F12 supplemented with 1% N2 (GIBCO), 2% B27 (GIBCO), 100 µg mL^−1^ penicillin/streptomycin, and 50 ng mL^−1^ BDNF (Proteintech). VPA (1 mm) was added to the differentiation medium after 2 d, and the medium was changed every 2 d.

### 2.7 Coculture of SGN and ACN Single Cells

Following the resuspension of the SGNs and ACNs in the plating medium. A droplet of 20 µL of cell suspension comprising either SGNs or ACNs was delicately deposited on each side of the PDL‐coated substrates, and the two droplets were separated approximately 1 mm in distance. The substrates were then placed in an incubator for 20–30 min to facilitate the initial phase of cell adhesion. Subsequently, an adequate volume of plating medium was introduced, and after a lapse of 3 h, it was substituted with a differentiation medium. The medium was changed every 2 d.

### 2.8 Generation of Human Neural Spheroids of the Auditory System

The auditory cortex tissue and cochleae from PCW 10–13 aborted fetuses were dissected and collected in ice‐cold PBS. The cochleae were dissected, and the epithelial and bony tissue were removed to purify the SGN regions. The SGN tissues were incubated in 0.125% trypsin‐EDTA for 15 min at 37 °C in the incubator. The auditory cortex tissues were digested for 7 min at 37 °C. Digestion was terminated by adding an equal volume of 10% FBS in the DMEM/F12 medium. Then, the SGN and cortex tissues were washed twice with DMEM and gently triturated with a 200 µL pipette. The cells were suspended in DMEM/F12 medium supplemented with 1% N2, 2% B27, 100 µg mL^−1^ penicillin/streptomycin, 50 ng mL^−1^ EGF (Proteintech), 50 ng mL^−1^ bFGF (Proteintech), and 50 ng mL^−1^ IGF (Proteintech). Cells (1 × 10^4^ cells per well) were seeded into an ultra‐low attachment 24‐well plate (Corning) to allow neural progenitor cells of each unit to proliferate. The culture medium was changed every 3 d.

After 6–7 d of suspension culture, the neural spheroids of each unit were subsequently transferred onto the PDL‐coated substrates for differentiation. To establish the coculture model with peripheral and central neural spheroids, a pair of SGN and ACN spheroids was placed on the substrates, ensuring that the distance between the two spheroids was approximately 300–400 µm. After full adherence, the differentiation medium was added. Then, 30 µL of Matrigel was added on differentiation Day 5 to cover the spheroids. The medium was replaced every 3 d.

### 2.9 Viral Labeling

The neural spheroids were transferred into a 96‐well ultralow attachment plate (Corning) on expansion Day 5. Each well contained 1–2 spheroids. The co‐cultured neural spheroids on TCPS and SA‐CNTs were incubated in 48‐well plates. The virus used for this study were AAV2/DJ‐CMV‐mCherry (Obio Technology Corp) and AAV2/9‐hSyn‐EGFP (PackGene Biotech). Viral titer ≥ 1E+13 GC mL^−1^. The virus diluted in the medium (1:100, v/v) was added to the target culture and incubated for 24 h. Fresh medium was replaced on the following day. Fluorescence was observed under a Leica DMi8 microscope with 488 and 555 nm excitation wavelength.

### 2.10 Cell Viability Assay

Cell viability was assessed by the Live/Dead staining kit (Thermo Fisher Scientific). The cells were incubated with calcein‐AM (green, an indicator of live cells), propidium iodide (red, an indicator of dead cells), and Hoechst (blue, an indicator of nucleus) in the differentiation medium (1:1000, v/v, respectively) at 37 °C for 10 min and then washed with PBS. Images were taken under a Leica DMi8 microscope with a 488/570 nm excitation wavelength. The number of live and dead cells was counted using ImageJ (National Institute of Health, USA).

### 2.11 Immunofluorescence Staining

The samples were fixed in 4% paraformaldehyde (PFA) at room temperature for 1 h. After washing three times with PBS, the samples were incubated in PBS with 1% Triton X‐100 and 10% FBS for 2 h at room temperature for blocking and permeabilization. The samples were incubated with primary antibodies diluted in PBS with 1% Triton X‐100 overnight at 4 °C. The primary antibodies used for staining are listed in **Table** **1**.

**Table 1:** List of antibodies.

ANTIBODIES	HOST SPECIES	SUPPLIER	IDENTIFIER
Tuj1	Mouse	Biolegend	801202
NF‐H	Chick	Abcam	ab72996
MAP2	Rabbit	Cell Signaling Technology	4542
Synapsin 1	Rabbit	Cell Signaling Technology	5297
Synaptophysin	Mouse	Abcam	ab309493
PSD95	Rabbit	Abcam	ab18258
Nestin	Mouse	Santa Cruz Biotechnology	sc‐23927
Sox2	Goat	R&D system	AF2018
KI67	Mouse	Cell Signaling Technology	9449
Sox9	Rabbit	Abcam	ab185966
MYT1	Rabbit	Proteintech	26204‐1‐AP
PAX6	Rabbit	Abcam	ab195045
SOX10	Rabbit	Abcam	Ab155279

After washing with PBS three times, the samples were incubated at room temperature with Alexa 488‐, 555‐, or 647‐conjugated anti‐rabbit, anti‐mouse, or anti‐goat secondary antibodies (Invitrogen) diluted at 1:400 in PBS with 1% Triton X‐100 for 6 h. After three washes in PBS, the samples were stained with DAPI (Sigma) for 10 min at RT. All the samples were kept in a light‐proof box during immunofluorescence staining. Z‐stacks of optical sections were captured on an Sp8 confocal microscope (Leica) and processed with ImageJ.

### 2.12 Optogenetics and Calcium Imaging

SGN spheroids were infected with AAV2/DJ‐CAG‐ChrimsonR‐Scarlet on expansion Day 5 and AAV2/DJ‐CAG‐GCaMP6s was applied to ACN spheroids. Then the SGN and ACN organoids were cocultured for another 30 d for differentiation. SA‐CNTs with co‐cultured organoids were placed in a 35 mm Glass Bottom Dish (Thermo Scientific) and live‐imaged using a 20x objective in a confocal microscope (CSU‐W1‐SoRa, Nikon) under environmentally controlled conditions (37 °C, 5% CO_2_). ChrimsonR was activated with 590 nm light (20s apart) for optogenetic stimulation. To record calcium spikes, GCaMP6s was imaged at a frame rate of 1.5 frames s^−1^. Stimulation experiments were sustained for at least 10 min. Single cells were carefully outlined in a region of interest (ROI), and intensity of fluorescence was measured over the entire time series by Imaris. The fluorescence change was defined as Δ*F*/*F*_0_ = (*F*_t_‐*F*_0_)/*F*_0_, where *F*_0_ was defined as the baseline fluorescence for each time point. The spiking traces were plotted in MATLAB.

### 2.13 Electrophysiology

The cultured SGNs were visualized through a 60X water‐immersion objective in an upright microscope (Olympus), and patch‐clamp recordings were made through an EPC10/2 amplifier (HEKA Electronics, Lambrecht Pfalz, Germany) driven by a PC computer running Patchmaster (HEKA Electronics). Recording pipettes were pulled from borosilicate glass capillaries (Sutter) and filled with internal solutions. For EPSC and induced AP recording, SGNs grown on TCPS or SA‐CNTs were immersed in an oxygenated extracellular solution containing (in mm) 135 NaCl, 5 KCl, 2 CaCl_2_, 1 MgCl_2_, 10 HEPES, and 10 D‐glucose (pH 7.30, osmolarity 300 mOsm). For outward K^+^ current recording, an additional 0.2 mm CdCl_2_ was included. For Na^+^ current recording, an additional 10 mm TEA‐Cl and 2 mm 4‐AP were included.

For EPSC, induced AP recording and outward K^+^ current recording, an internal solution containing (in mm) 135 K‐methane sulfonate, 20 KCl, 2 EGTA, 3 Mg‐ATP, 10 HEPES, 0.5 Na‐GTP (pH 7.20, osmolarity 300 mOsm). A Cs+‐based internal solution was used to block the K^+^ current and isolate the Na^+^ current. For Na^+^ current recording, pipettes were filled with an internal solution containing (in mm): 135 Cs‐methane sulfonate, 10 CsCl, 10 TEA‐Cl, 10 HEPES, 2 EGTA, 3 Mg‐ATP and 0.5 Na‐GTP (pH 7.20, osmolarity 300 mOsm). Cells were all held at –90 mV, and the uncompensated series resistance had a typical value of 6–10 MΩ. All patch‐clamp experiments were performed at room temperature, and the liquid junction potential (≈10 mV) was corrected offline.

*Bhlhb5*‐tdTomato+ SGNs were elected from mice to perform whole‐cell patch clamp experiments. Outward K^+^ currents and Na^+^ currents were recorded under voltage clamp mode.

AAV2/9‐hSyn‐EGFP was used to label induced human SGNs and selected GFP+ cells to perform whole‐cell patch clamp experiments. Outward K^+^ currents were recorded under the voltage‐clamp mode, and induced APs and EPSCs were recorded under the current‐clamp mode. A homemade macro written in Igor Pro 6.22 was used for the analysis of K^+^ currents and Na^+^ currents. Statistical analyses were performed in GraphPad Prism 7.

### 2.14 Statistical Analysis

All experiments were performed at least three times. The data were analyzed with GraphPad Prism 7. Statistical analysis was performed by unpaired two‐tailed Student's t test or Mann‐Whitney test when two groups were compared. The results are presented as the means ± SEMs, and the statistical significance was assigned as *P < 0.05, **P < 0.01, ***P < 0.001, and ****P < 0.0001, respectively.

## 3 Results

### 3.1 The Design of a Carbon Nanotube‐Based System for the Integration of Auditory Neural Circuits

**Figure 1:**
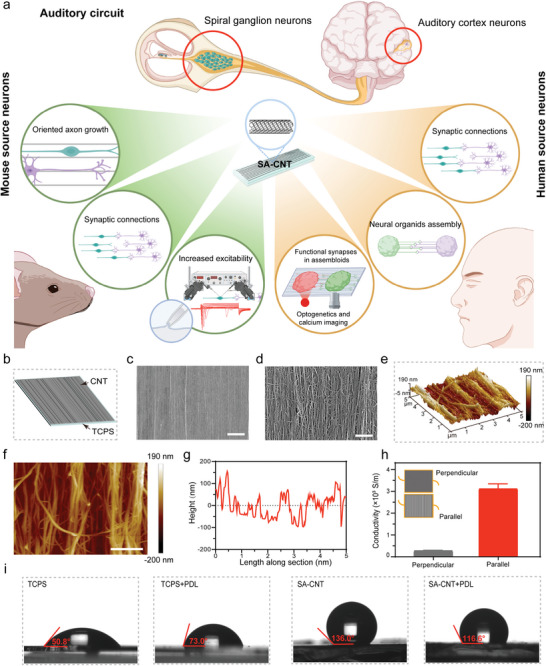
SA‐CNT‐based system for the integration of auditory neural circuits. a) Schematic representation of auditory pathway, including SGNs in the inner ear and ACNs in the temporal lobe for acoustic perception (upper). Schematic depicting the generation of auditory neural circuit model with mouse source neurons (lower left) and human source neurons (lower right). b) Schematic representation of SA‐CNTs. c,d) Representative SEM images of SA‐CNT at c) lower and d) higher magnifications. Scale bar: c) 20 µm, d) 2 µm. e–g) Representative e) 3D and f) 2D AFM surface topography micrographs of SA‐CNT. Scale bar, 1 µm. g) Related section analysis profiles of SA‐CNT. h) The conductivity of the SA‐CNT. i) Representative images of the water contact angle on different substrates.

Inspired by the precise projection of the SGN in the auditory cortex through nuclei, the acoustic information received by HCs is transmitted to central neurons for perception (**Figure** **1**a). We recognized the urgent need for materials to enhance the oriented growth of neural axons to facilitate the establishment of effective synaptic connections of the auditory neural circuit model. We introduced SA‐CNTs to culture single primary mouse and human bipolar SGNs and multipolar ACNs, which rendered the directional growth of the two neurites on the patterned substrates (Figure 1a). The substrates increased the potassium ion activity of mouse SGNs and drove SGNs to project to ACNs to form physiological connections. In a SA‐CNT‐Matrigel‐based 3D system, functional synaptic connections were established between SGNs and ACNs from human peripheral and central neural spheroids.

We integrated SA‐CNT arrays onto polystyrene (PS) substrates (Figure 1b), and the surface morphology of SA‐CNT was observed using scanning electron microscopy (SEM). As depicted in the SEM image at lower magnification, the SA‐CNT array exhibits uniformity over a large area (Figure 1c). Upon closer examination of the high‐magnification SEM image, it was observed that the carbon nanotubes were aligned in the same direction, with uniform spacing between neighboring SA‐CNTs (Figure 1d). Subsequently, an atomic force microscope (AFM) was employed to further scrutinize the surface topography of the SA‐CNTs. The results demonstrated the formation of a groove topology with a height of approximately 100 nm on the surface of SA‐CNT (Figure 1e–g). Neural cells constitute a class of cells characterized by electrical activity, encompassing processes such as neuronal differentiation and maturation, all intricately linked to electrical phenomena. Consequently, substrate conductivity plays a pivotal role in the regulation of nerve cells. Hence, we further investigated the electrical conductivity of SA‐CNTs, revealing significantly higher conductance in parallel measurements compared to perpendicular measurements (Figure 1h). Finally, to enhance cell adhesion to the substrate, a layer of poly‐D‐lysine (PDL) was applied to coat the substrate before cell seeding. Subsequent measurement of the hydrophilicity of different substrates through water contact angle testing suggested an improvement in the hydrophilicity of SA‐CNT after coating with PDL, thereby facilitating the promotion of cell adhesion (Figure 1i).

### 3.2 Carbon Nanotubes Retain Primary Mouse SGNs and ACNs Survive

**Figure 2:**
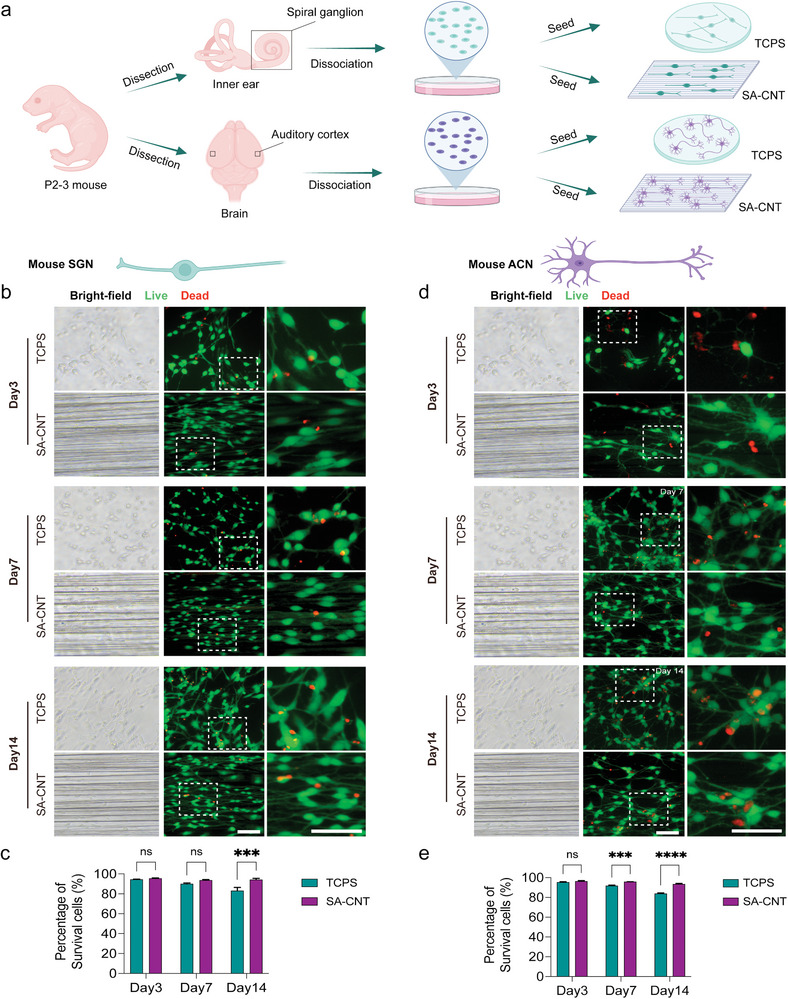
SA‐CNT maintains primary mouse auditory neuron survival. a) Schematic depicting the experimental strategy for culturing murine SGNs and ACNs on TCPS and SA‐CNTs. Fluorescence and bright‐field images showing live/dead staining of b) SGNs and d) ACNs cultured on TCPS and SA‐CNTs on Days 3, 7, and 14. The entire cytoplasm of the live cell was labeled with calcein‐AM (green), and nuclei of the dead cell were labeled with propidium iodide (red). Scale bar, 100 µm. The proportion of live cells in c) SGN and e) ACN culture. 5–6 fields at each time point, three independent experiments. The data are presented as the mean ± SEM, ***P < 0.001, ****P < 0.0001 (student's t test).

To assess the biocompatibility of SA‐CNTs for primary auditory neurons, mouse SGNs and ACNs were attached to the materials and tissue culture polystyrene (TCPS) for culture (**Figure** **2**a). Fresh spiral ganglia of the cochlea and primary auditory cortex were dissected from postnatal (P) 2–3 wild‐type (WT) mice and then dissociated into single cells using enzyme digestion. Cells from the two different tissues were cultured for up to half a month to observe the biocompatibility of SA‐CNTs.

We used calcein AM staining (green) to identify the live cells and propidium iodide (red) to label the dead cells. On Day 3, the mean rates of surviving mouse SGNs cultured on the TCPS and SA‐CNTs were 94.39% and 95.37%, respectively (Figure 2b,c). As the cultivation time progressed, more dead cells were found in the TCPS group. The survival rate of SGNs was significantly better on SA‐CNTs than on TCPS, as 83.14% survived SGNs were found in the TCPS group and 94.23% survived SGNs were retained in the SA‐CNT cultured group on Day 14. This gap also exists in central auditory neurons as the culture time increases, and it can be seen that the neuronal viability on SA‐CNTs is significantly higher than that in TCPS even on Day 7 (Figure 2d,e). On Day 7, the mean surviving mouse ACN on the TCPS and CNT was 91.72% and 95.68%, respectively. On Day 14, a 9.50% ± 0.92% gap was detected between the groups, and the mean surviving mouse ACN on the TPCS and SA‐CNT was 83.90% and 93.40%, respectively. The results suggested that SA‐CNT retains primary mouse auditory neurons and shows good biocompatibility with auditory neurons.

### 3.3 Oriented Growth of Single Mouse SGNs and ACNs

**Figure 3:**
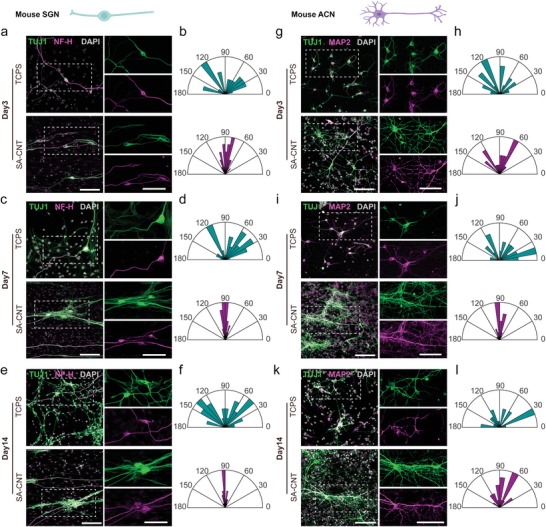
SA‐CNT directs oriented axon outgrowth of mouse peripheral and central auditory neurons. Representative confocal images show TUJ1 and NF‐H staining of SGNs cultured on TCPS and SA‐CNTs on a) Day 3, c) Day 7, and e) Day 14. Scale bar: 100 µm. Orientation angle distribution of SGN neurites on b) Day 3, d) Day 7, and f) Day 14. Representative confocal images show TUJ1 and MAP2 staining of ACNs cultured on TCPS and SA‐CNTs on g) Day 3, i) Day 7, and k) Day 14. Scale bar, 100 µm. Orientation angle distribution of ACN neurites on h) Day 3, j) Day 7, and l) Day 14.

Oriented projections of SGN axons were essential for the circuitry between the cochlear sensory organ and the central auditory cortex,^[^ ^13^ ^]^ and we then detected the effect of aligned SA‐CNTs on the growth pattern of auditory neurons. β‐Tubulin III (TUJ1)‐labeled SGNs mainly presented as bipolar neurons, and neurons were stained with neurofilament heavy chain (NF‐H) to trace the axons (**Figure** **3**a). On Day 3, SGNs grew in a disorderly manner on the TCPS, while SGNs exhibited a certain parallel growth pattern on SA‐CNTs. To precisely measure the direction of axon growth, we built a vertical line perpendicular to the long axis of the neuron body and then measured the angle between the axon and the vertical line. As shown in Figure 3b, the measured angles of SGNs on the TCPS showed a large span from 19.9° to 167.7°, and the measured angles of SGNs on SA‐CNTs ranged from 46.4° to 115.6°. As the culture time progressed, SGNs appeared even more disorganized on the TCPS, especially on Day 14 (Figure 3c–f). On Day 14, the SGNs conspicuously formed an oriented growth pattern parallel to the SA‐CNT texture, as the measured angles of SGNs on SA‐CNTs ranged from 66.1° to 120.1° (Figure 3e,f). The measured angles of SGNs on the TCPS ranged from 6.2° to 167.1° on Day 14, which exhibited a gap of 160.9° ± 8.5° and was much larger than the 54.0° ± 2.3° gap on SA‐CNTs.

We further assessed the growth pattern of ACNs with multipolar axons to test whether SA‐CNTs would manipulate central neuron projections. Surprisingly, multipolar axons on SA‐CNTs showed a relatively oriented growth pattern parallel to the SA‐CNT texture compared to TCPS, even on Day 3 (Figure 3g,h). During the entire cultivation process of approximately half a month, the axons of central auditory neurons maintained an oriented growth direction parallel to the SA‐CNTs, while the axons on the TCPS grew randomly (Figure 3g–k). On Day 14, the measured angles of ACNs on the TCPS showed a large span from 0° to 178.3°, while the measured angles of ACNs on SA‐CNTs ranged from 35.4° to 147.3° (Figure 3l). These results suggest that SA‐CNTs guide the oriented growth of axons of both mouse peripheral and central auditory neurons.

### 3.4 Establishment of Mouse Auditory Circuits by Coculturing SGNs and ACNs on SA‐CNTs

**Figure 4:**
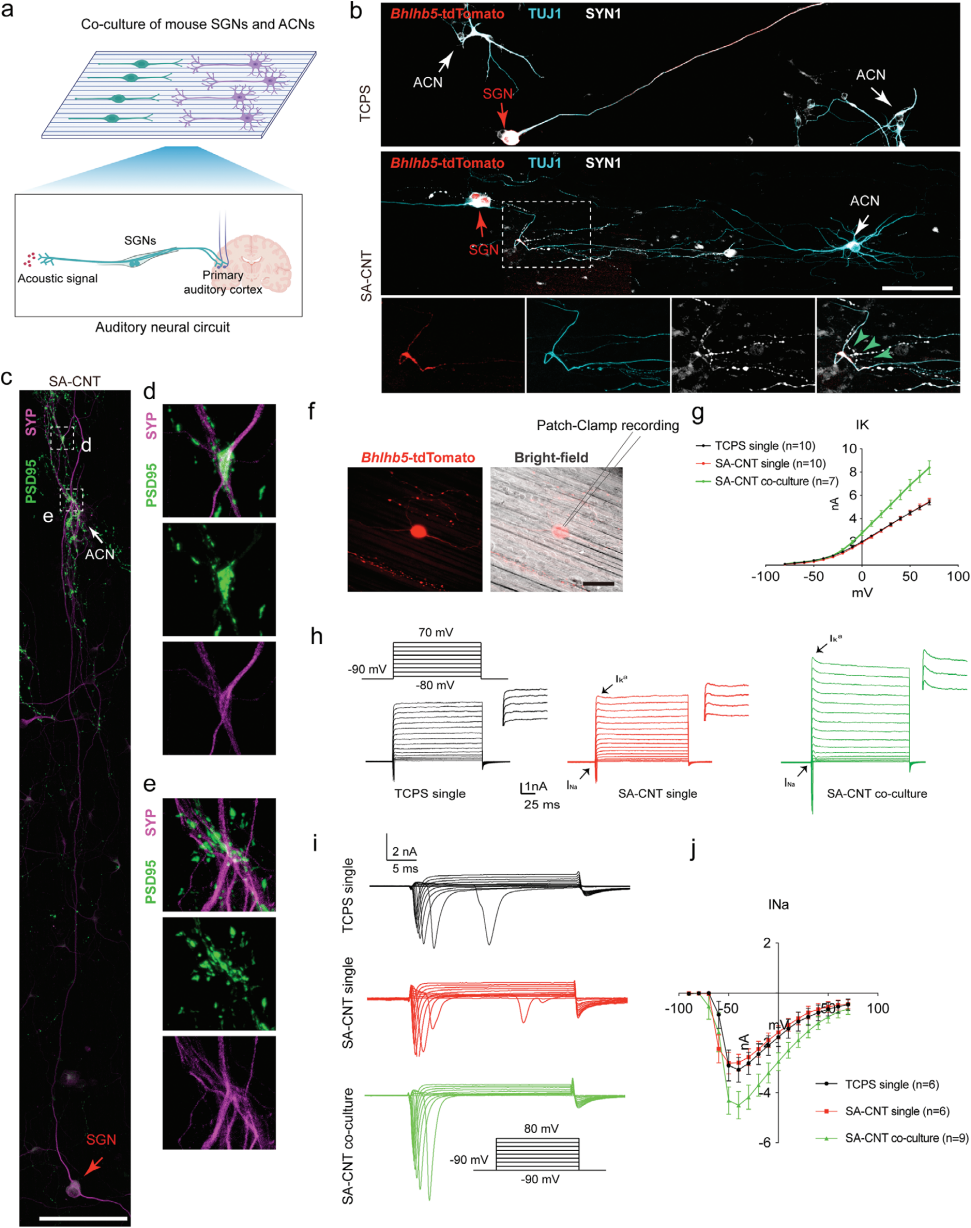
Generation and electrophysiological characteristics of mouse auditory circuits. a) Schematic representation of the mouse SGNs and ACNs on SA‐CNT based coculture system. b) Confocal images of the co‐culture system stained with TUJ1 and SYN1 on Day 15. The red arrows indicate *Bhlhb5*‐tdtomato traced SGNs. The white arrows indicate wild‐type ACNs. The green arrows indicate SYN1+ presynaptic vesicles. Scale bar, 100 µm. c) Confocal images of the SA‐CNT‐based coculture system stained with PSD95 and SYP on Day 15. The red arrows indicate SGNs. The white arrows indicate ACNs. Scale bar, 100 µm. d,e) Higher magnification of the intersected region showing the SYP+ presynaptic vesicles co‐localized with PSD95+ postsynaptic vesicles. f) Fluorescence and bright‐field images showing recording pipettes patched to *Bhlhb5*‐tdTomato+ SGNs. Scale bar, 50 µm. g) Mean ± SEM peak current–voltage relations of outward potassium currents for 10 SGNs in the TCPS group (black), 10 SGNs in the SA‐CNT group (red) and 7 SGNs in the SA‐CNT coculture group (green). h) Family of outward potassium currents recorded from three SGNs from TCPS (black), SA‐CNT (red), and SA‐CNT coculture groups, evoked by the series of voltage steps shown below. i) Families of Na^+^ current activation from three SGNs from TCPS (black), SA‐CNT (red), and SA‐CNT coculture groups, evoked by the series of voltage steps shown below. j) Mean ± SEM peak current‐voltage relations of Na^+^ current activation for 6 SGNs in the TCPS group (black), 6 SGNs in the SA‐CNT group (red) and 9 SGNs in the SA‐CNT coculture group (green).

To mimic mouse auditory neural circuits in vitro, we cocultured single SGNs and ACNs to form synapses between the two types of neurons (**Figure** **4**a). To distinguish SGNs and ACNs, SGNs were harvested from *Bhlhb5*‐tdTomato mice, while ACNs were obtained from WT mice. As shown in Figure 4b, we rarely detected tdTomato+ SGNs physically interacting with multipolar ACNs in the TCPS co‐culture system. However, we noticed that the oriented tdTomato+/TUJ1+ SGN axon formed a connection with the axonal terminals of ACNs in the SA‐CNT system on Day 15. The synaptic protein recombinant synapsin I (SYN1) was found to be expressed in the ACN axonal terminals in close contact with SGN axonal terminals (Figure 4b). In addition, we noticed postsynaptic marker postsynaptic density (PSD)95 adjacent to the ACN synaptic protein synaptophysin (SYP) (Figure 4c–e), suggesting the establishment of synaptic connectivity of auditory neurons in the SA‐CNT system.

We further conducted electrophysiological recordings to precisely assess the electrical properties of the SGNs in the neural circuits, and a patch clamp was used to record the electrical activity of *Bhlhb5*‐tdTomato+ SGNs (Figure 4f). Various ion channels are crucial in the function of neurons and reflect the state of neuronal cells.^[^ ^14^ ^]^ We examined voltage‐gated K current (IK) in tdTomato+ cells, and the presence of fast‐inactivating K^+^ channels mediating currents (IKA) was detected (Figure 4h). *I*–*V* curve analysis showed that there was no significant difference in the size of outward potassium currents between the TCPS group and SA‐CNT group, but a conspicuously larger size of outward potassium currents was found in the coculture group (Figure 4g). However, we observed the disappearance of IKA in the TCPS group, which suggests that some ion channels degraded after a long period of culture. Interestingly, we observed significant IKA in the SA‐CNT group, indicating that SA‐CNT has a certain effect on maintaining the properties of neuronal IKA channels, which have been proven to be involved in many physiological functions, including membrane excitability regulation and the control of the firing pattern.^[^ ^15^ ^]^ *I*–*V* curve analysis showed that there was no significant difference in the size of the outward potassium current between the TCPS group and SA‐CNT group, but the outward potassium current was significantly larger in the co‐culture group (IK at 0 mV, TCPS group, IK = 2.016 ± 0.054 nA, SA‐CNT group, IK = 1.931 ± 0.098 nA, coculture group, IK = 2.750 ± 0.196 nA).

We next examined voltage‐gated Na current (INa), which determines neuronal properties, including action potential (AP) generation and the local depolarization of neurons.^[^ ^14^ ^]^ To isolate INa currents, we used a Cs+‐based internal solution and added 10 mm TEA‐Cl and 2 mm 4‐aminopyridine to the external solution to block voltage‐gated K^+^ currents. In addition, we added 0.2 mm CdCl_2_ to the external solution to block voltage‐gated Ca^2+^ currents (ICa). We observed Na currents with a voltage step from ‐90 to 70 mV, with a step of 10 mV (Figure 4i). As shown in the current–voltage (*I*–*V*) curve (Figure 4j), INa recorded from SGNs on both TCPS and SA‐CNT groups was activated rapidly at approximately ‐60 mV and peaked at ‐40 mV. Although no obvious difference was found in the peak amplitude between the TCPS and SA‐CNT groups, significantly larger peak amplitudes of INa currents were found in the co‐culture group (INa peak, TCPS INa = −3.197 ± 0.523 nA; SA‐CNT, INa = −3.103 ± 0.359 nA; coculture, INa = −4.903 ± 0.493 nA). Taken together, the results indicate that SA‐CNTs maintain the ion excitability of auditory neurons to a certain extent, and mouse SGNs and ACNs achieved synaptic connections and ion channel interactions on SA‐CNTs.

### 3.5 Neural Circuit Formation between Human SGNs and ACNs on SA‐CNTs

**Figure 5:**
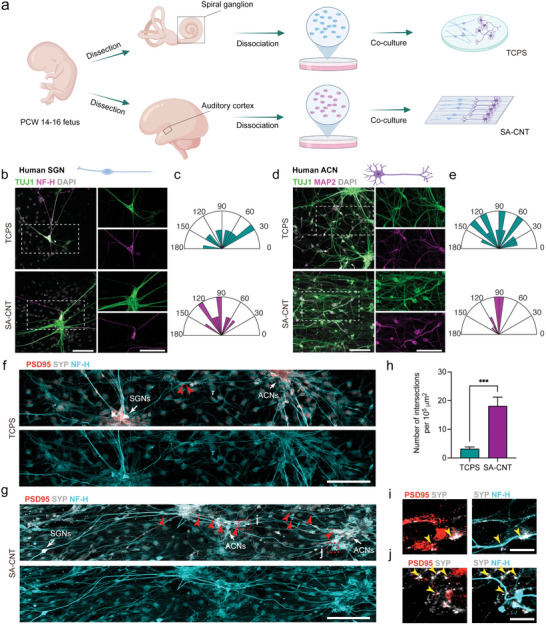
Generation of human auditory circuits by coculturing primary SGNs and ACNs. a) Schematic depicting the experimental strategy for culturing human SGNs and ACNs on TCPS and SA‐CNTs. b) Representative confocal images show TUJ1 and NF‐H staining of human SGNs cultured on TCPS and SA‐CNTs on Day 14. Scale bar, 100 µm. c) Orientation angle distribution of human SGN neurites. d) Representative confocal images show TUJ1 and MAP2 staining of human ACNs cultured on TCPS and SA‐CNTs on Day 14. Scale bar, 100 µm. e) Orientation angle distribution of human ACN neurites. f,g) Representative confocal images show PSD95, SYP, and NF‐H staining of the human SGNs and human ACNs co‐cultured on f) TCPS and g) SA‐CNT on Day 20. The white arrows indicate SGN and ACN respectively. The red arrowheads indicate cellular intersections. Scale bar, 100 µm. h) The average number of cellular intersections per 10^5^ µm^2^. The data are presented as the mean ± SEM, ***P < 0.001 (student's t test). i,j) Higher magnification of the selected region shows the SYP+ presynaptic vesicles co‐localized with PSD95+ postsynaptic vesicles along the NF‐H+ neurites. Scale bar, 10 µm.

As primary human cultured cells are closer to the characteristics of human tissue in vivo than cell lines or mouse cells, and few coculture models for human auditory neurons currently exist, we tested the behavior of human auditory neurons on the SA‐CNT system and attempted to establish a human auditory circuit model in vitro. Inner ear spiral ganglion tissues and primary auditory cortex were obtained from aborted human fetuses ranging from post‐conception week (PCW) 14‐16, then disassociated into single cells and cultured on TCPS and SA‐CNTs (**Figure** **5**a). As shown in Figure 5b, TUJ1/NF‐H‐labeled SGNs exhibited a pattern of directional growth on SA‐CNTs, and the axons grew parallel to the SA‐CNT patterns on Day 15. Compared to the range of the measured angles from 30.6° to 170.5° on TCPS, SGN axons on SA‐CNTs exhibited a smaller range from 45.3° to 148.0° (Figure 5c). The oriented growth of axons on the aligned SA‐CNTs was also found in human ACNs, as the gap of measured angles of axons was obviously smaller on SA‐CNTs than on TCPS (TCPS: 140.9 ± 11.2°, SA‐CNT: 54.2 ± 5.3°) (Figure 5d). The results showed that aligned SA‐CNTs promote oriented growth of primary human neurons from both peripheral and central auditory systems.

To test whether the aligned SA‐CNTs would facilitate synapse formation between single human SGNs and ACNs, similar to mouse source neurons, we co‐cultured the two kinds of human neurons on the separated sides of the TCPS and SA‐CNT. On Day 20, all the nerves were labeled with NF‐H, the bipolar neurons were identified as human SGNs, and the multipolar neurons were identified as human ACNs (Figure 5f,g). We detected a few SGN axons physically interacting with multipolar ACNs in the TCPS co‐culture system (Figure 5f). In contrast, axons of SGNs and ACNs were relatively parallel arranged, and more physical contacts were detected between the two kinds of auditory neurons in the SA‐CNT co‐culture system (Figure 5g). The number of SGN‐ACN axonal intersections was significantly increased in the SA‐CNT group compared to the TCPS group (TCPS: 3.19 ± 0.64 per 10^5^ µm^2^, SA‐CNT: 18.13 ± 3.10 per 10^5^ µm^2^) (Figure 5h). The synaptic proteins SYP and PSD95 localized in pairs at axonal intersections on SA‐CNTs, while paired localization was rarely found in TCPS (Figure 5f,g). As shown in Figure 5i,j, the localization of synaptic proteins in the SA‐CNT group suggested that SGNs formed synaptic connections with ACNs. Taken together, the human neural circuit of the auditory system was established based on the SA‐CNT scaffold.

### 3.6 Integration of Human Peripheral and Central Auditory Neural Organoids

**Figure 6:**
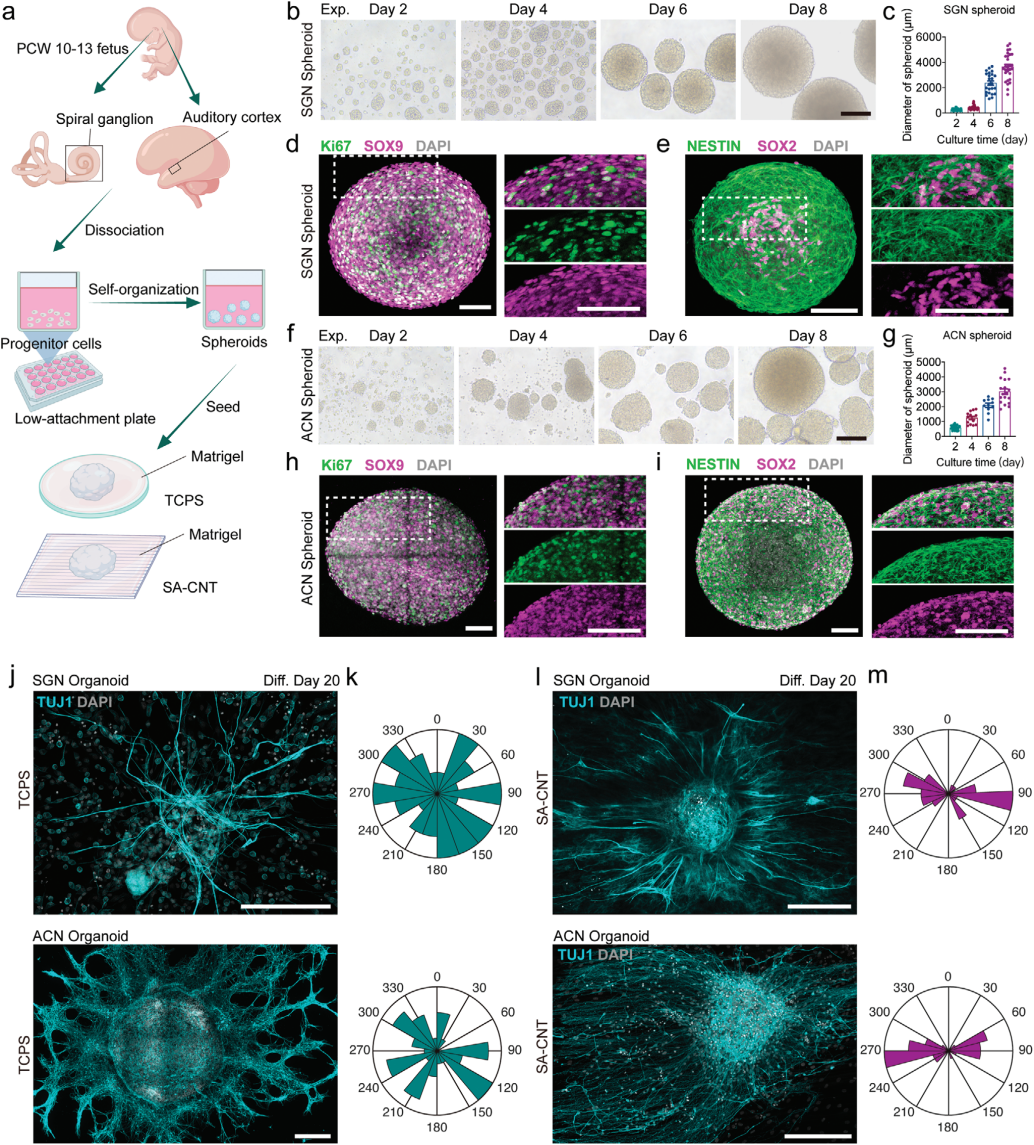
Generating human neural organoids from primary neural progenitor cells. a) Schematic representation of the experimental strategy for generating human neural spheroids. b,f) Bright‐field images of human neural spheroids derived from b) SGN progenitor cells and f) ACN progenitor cells on Days 2, 4, 6, and 8. Scale bar, 100 µm. c,g) Quantification of the size of c) SGN and g) ACN derived spheroid on different days in vitro. Each dot represents a spheroid. The data are presented as means ± SEM. d) Representative confocal images showing the expression of Ki67 and SOX9 in SGN spheroids on Day 7. e) Representative confocal images showing the expression of NESTIN and SOX2 in SGN spheroids on Day 7. h) Representative confocal images showing the expression of Ki67 and SOX9 in ACN spheroids on Day 7. i) Representative confocal images showing the expression of NESTIN and SOX2 in ACN spheroids on Day 7. Scale bar, 100 µm. j,l) Representative confocal images show TUJ1 staining of the SGN and ACN spheroid differentiated on j) TCPS and l) SA‐CNT. k,m) Orientation angle distribution of neurites derived from SGN and ACN spheroids differentiated on k) TCPS and m) SA‐CNT. Scale bar, 200 µm.

As organoids recapitulate the structural and functional characteristics of target organs better than single cells, we aimed to utilize primary progenitor cells to construct synaptic connections between the auditory central organoids and peripheral organoids in the SA‐CNT system. SGN and ACN progenitor cells were isolated from aborted human fetuses ranging from PCW 10‐13 and then proliferated into spheroids in suspension culture (**Figure** **6**a). Two kinds of spheroids were cocultured in the SA‐CNT‐Matrigel 3D culture system to differentiate into neural organoids and induce the respective axons to grow directionally to form connections. Spheroids were formed from either the SGN or ACN progenitor cells, and the size of spheroids increased progressively (Figure 6b,c,f,g). During the expansion stage, a substantial presence of neural progenitor cells, characterized by SOX9 and NESTIN expression, was evident in both central and peripheral neural spheroids. Additionally, these cells exhibited positivity for the proliferative marker Ki67 (Figure 6d,h, Figure S1b,d, Supporting Information). In contrast to NESTIN and SOX2, which displayed near‐complete co‐labeling in central spheroids, only a subset of NESTIN+ progenitor cells coexpressed SOX2 in peripheral spheroids, suggesting distinct genetic attributes between SGN and ACN progenitor cells (Figure 6e,i). Moreover, our findings revealed the presence of MYT1+ SGN progenitors and SOX10+ glial cell progenitors^[^ ^16^ ^]^ within SGN spheroids, whereas PAX6+/NESTIN+ neural progenitor cells predominated in ACN spheroids (Figure S1c,e,f, Supporting Information). Remarkably, only a sparse population of cells expressing the mature neural marker MAP2 was observed in either SGN or ACN spheroids (Figure S1a, Supporting Information).

The two kinds of neural spheroids were transferred to adhere to the TCPS and SA‐CNTs on proliferation Day 7 for further neuron induction, and a droplet of Matrigel was added to form a 3D microenvironment. On differentiation Day 20, immunocytochemistry showed neural organoids with TUJ1+ neurons in the central region with extended axons (Figure 6j,l). As shown in Figure 6k,m, the axons of both SGN organoids and ACN organoids that grew on SA‐CNTs were parallel to the scaffold patterns, while the axons on TCPS grew outward randomly. Furthermore, we noted an increase in the TUJ1+ cell population within the differentiated organoids in the SA‐CNT groups. The proportion of TUJ1+ cells in the organoids from the TCPS group measured 22.78±1.49%, contrasting with the SA‐CNT group, where this proportion escalated to 41.11 ± 2.90%. Similarly, SA‐CNTs demonstrated the ability to enhance ACN organoid differentiation (Figure S1g, Supporting Information). Overall, we generated human neural region‐specific organoids of peripheral and central systems from primary neural progenitor cells, and SA‐CNTs promoted neural organoid differentiation.

### 3.7 Functional Synaptic Connections in Auditory Organoid‐Integrated Neural Circuits

**Figure 7:**
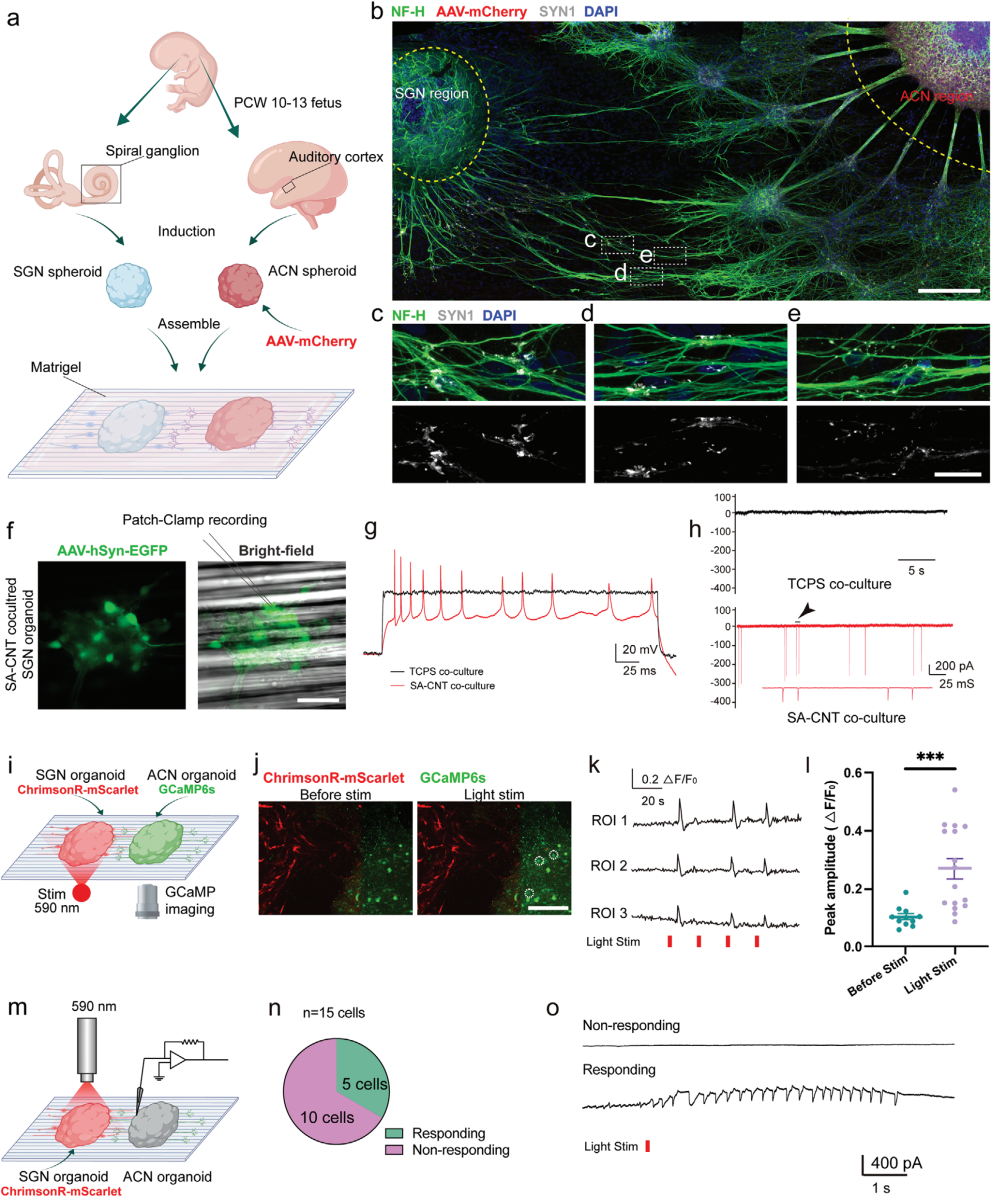
The morphological and electrophysiological characteristics of the human auditory organoid‐integrated neural circuits. a) Schematic representation of the experimental strategy for integrating SGN and ACN spheroids by 3D coculture. b) Representative confocal images show TUJ1 and SYN1 staining of the human SGN and mCherry+ ACN spheroids cocultured on SA‐CNT on Day 20. Scale bar, 200 µm. c–e) Higher magnification of the selected region showing the SYN1+ synaptic vesicles. Scale bar, 20 µm. f) Fluorescence and bright‐field images showing recording pipettes patched to cocultured SGN organoid on Day 30. SGNs transfected with AAV 2/9‐hSyn‐ EGFP. Scale bar, 50 µm. g) Representative spontaneous spikes recorded from SGNs in the SA‐CNT group (red) but not in the TCPS group (black), by holding the cells at zero current. h) Typical spontaneous excitatory postsynaptic currents (EPSCs) recorded from two SGNs cultured on TCPS (black) and SA‐CNT (red). i) Schematic detailing optogenetic stimulation coupled with GCaMP imaging in cocultured SGN and ACN organoids. j) SGN organoid responding to light stimulation, showing changes in GCaMP6s signals (green) before stimulation (before stim) and after stimulation (light stim). Scale bar, 200 µm. k) Representative traces of calcium imaging from three ROIs. ROI, region of interest. l) Peak amplitudes of Δ*F*/*F*_0_ in ACN organoids after light stimulation. 11 ROIs in Before Stim and 16 ROIs in Light Stim. The data are presented as the mean ± SEM, ***P < 0.001 (Mann Whitney test). m) Schematic diagram illustrating the method for whole‐cell patch clamp recording with optogenetic activation. SGN organoids expressed Chrimson‐mScarlet. n) The proportion of ACNs that responded after using 590 nm red light to induce AAV‐transfected SGNs. 15 patched ACN cells. o) Representative current responses observed in ACNs after stimulating SGNs with 590 nm light. The upper shows non‐responding cells and the lower line shows responding cells.

We next cocultured the SGN and ACN spheroids on the SA‐CNTs, with Matrigel droplets applied to envelop the attached spheroids, thereby creating a 3D microenvironment (**Figure** **7**a). To track ACN progenitor cell‐derived spheroids, we introduced on AAV2/DJ‐CMV‐mCherry expansion Day 5. Subsequently, a pair of SGN spheroid and mCherry+ ACN spheroid was transferred to adhere to the SA‐CNTs. As illustrated in Figure 7b, after 20 d of co‐culture, neurites derived from mCherry‐ SGN organoids and mCherry+ ACN organoids extended toward each other, forming intersections on the SA‐CNTs. Importantly, the neural organoids extended elaborate neurites with fine dendritic structures revealed by TUJ1 labeling (Figure 7b), further corroborated by positive staining for NF‐H (Figure S2a, Supporting Information). Synaptic puncta, identified by staining for SYN1, were observed along TUJ1+ neurites (Figure 7b–d). Additionally, paired localization of SYP and PSD95 expression was detected along the intersections of NF‐H+ neurites derived from SGN organoids and ACN organoids (Figure S2a–d, Supporting Information).

Then, electrophysiological recordings were performed to assess the electrical properties of the differentiated SGNs in organoids. To trace SGNs in neural organoids, we transfected the co‐cultured organoids with AAV2/9‐hSyn‐EGFP on differentiation Day 14 (Figure 7f). EGFP+ SGNs were selected for the patch‐clamp study on differentiation Day 20. We first examined voltage‐gated K current (IK) with voltage steps from ‐80 to 70 mV, 10 mV a step (Figure S2e,f, Supporting Information). Normalized GV relations for outward potassium currents are shown in Figure S2h (Supporting Information) and fitted by a Boltzmann curve with *V*1/2 = 1.314 mV, *K* = 11.40 in the TCPS group, and *V*1/2 = 3.681 mV, *K* = 15.63 in the SA‐CNT group, which indicated that SA‐CNT enlarged the potassium currents of SGNs (Figure S2h, Supporting Information). Additionally, *I*–*V* curve analysis showed that there was a significant difference in the size of the outward potassium current between the TCPS group and SA‐CNT group (IK at 0 mV, TCPS group, IK = 0.469 ± 0.059 nA, SA‐CNT group, IK = 0.855 ± 0.061 nA, *p* = 0.0009) (Figure S2g, Supporting Information).

Next, we investigated the spiking pattern, which is an important indicator that directly reflects neuronal functions,^[^ ^14^ ^]^ in SGNs in co‐cultured organoids. We recorded spontaneous APs by holding cells at 0 pA under the current‐clamp mode (Figure 7g). Spontaneous APs were detected in some SGNs on SA‐CNTs (2 of 8), while no SGNs exhibited spontaneous spiking on TCPS (0 of 8). In addition, we applied step currents from 10 to 100 pA,10 pA a step, and recorded the spiking responses. We found that SGNs on SA‐CNT fired more spikes than SGNs on TCPS for a duration of 150 mS (Figure S2i,k, Supporting Information). Especially for step current injections of 40 to 100 pA, SGNs in the SA‐CNT group fired multiple spikes, while SGNs in the TCPS group only fired one spike (Figure S2j, Supporting Information). As shown in Figure 7h, excitatory postsynaptic currents (EPSCs) were detected only in the SA‐CNT group, indicating that SGNs in the peripheral organoids established synaptic connections with neurons in the central organoids.

To determine whether the of SGN organoids formed functional synaptic connections with the ACN organoids, we cocultured SGN organoids expressing opsin ChrimsonR‐Scarlet with ACN organoids expressing the calcium indicator GCaMP6s (Figure 7i). ChrimsonR is a light‐sensitive opsin, which enables the optical activation of the electrical activity in excitable cells.^[^ ^17^ ^]^ Calcium indicators, such as GCaMP6s, rely on rapid changes in intracellular free calcium to track neural activities.^[^ ^18^ ^]^ Subsequently, upon exposure to 590‐nanometer light to activate ChrimsonR‐Scarlet in the SGN organoids, we observed calcium responses in the ACN organoids 30 days post‐co‐culture (Figure 7j,k). Notably, the peak ΔF/F_0_ signal intensity for the neurons within the ACN organoids exhibited a significant increase following light stimulation (Figure 7l), indicative of functional connectivity between the SGN and ACN organoids. Meanwhile, through whole‐cell patch‐clamp experiments, we found that the application of 590 nm light stimulation in the SGN organoids induced currents in the ACN organoids (Figure 7m,o). 5 out of 15 cells were responsive to the stimulation (Figure 7n). Thus, both calcium imaging and electro‐physiological recording demonstrate the functional connectivity between the ACN organoids and the SGN organoids.

Taken together, these results show that SA‐CNT promotes human SGNs of peripheral neural organoids to mature in electrophysiological functions and form functional neural synapses with auditory neurons of central neural organoids.

## 4 Discussion

In this work, we established an auditory neural circuit model with functional synaptic connections by utilizing SA‐CNT substrates, step by step from the coculture of single primary mouse neurons to the coculture of human neural organoids. The directional projection of peripheral and central neurons of the auditory system guided by the aligned substrates promotes integrating the auditory neural circuitry, thereby paving the way for innovative methodologies for establishing connections between region‐specific neural units.

Synthetic scaffolds are designed with nanofibers, which hold aligned nanostructures and oriented patterns to orient cell and axon growth in a specific direction.^[^ ^19^ ^]^ Materials with nanofibers were shown to improve cell adhesion and differentiation and direct cells to self‐assemble.^[^ ^20^ ^]^ Among various biomaterials, carbon nanotubes (CNTs) have shown great potential for facilitating neuronal differentiation and driving axon growth.^[^ ^21^ ^]^ SA‐CNT in this work with a supper‐aligned pattern that directs the axons of bipolar SGNs growth parallel to the nanotubes, as well as the axons of multipolar neurons from the central auditory system, which highlights the powerful capacity to promote nerve directional growth of the scaffolds. Beyond studies limited to region‐specific neurons with the same source,^[^ ^19^, ^22^ ^]^ we cocultured central and peripheral neurons of the auditory system and realized functional synaptic connections on the scaffold. Apart from the low chemical reactivity,^[^ ^23^ ^]^ the directional growth pattern provides the opportunity for contact of the kinds of nerve endings. SA‐CNT substrates promote the cell viability might be attributed to generating physical influence on cellular adhesion. It is noteworthy that SA‐CNT advocates for the excitability of auditory neurons, potentially ascribed to its conductivity facilitating a conducive milieu within the ionic liquid culture system.^[^ ^24^ ^]^ Further studies should explore whether and how such mechanisms influence specific genes and proteins responsible for SA‐CNT‐induced neuron growth and synaptogenesis.

The electrophysiological characteristics of the cocultured mouse and human SGNs support that the peripheral‐central neural circuits are functional. In the cocultured single mouse neurons, enlarged voltage‐gated Na^+^ current and K^+^ current indicate active electrical activities in the coculture systems. As evidenced by the physical synaptic contacts, the ion channel data indicate the establishment of functional synaptic connections in the mouse circuit. Our results showed that close contact with SA‐CNT bears no appreciable difference in the voltage‐gated Na^+^ current of mouse SGNs but selectively contributes to the retention of IKA, which is involved in several physiological functions, including the maintenance of repetitive firing and the regulation of membrane excitability.^[^ ^15^, ^25^ ^]^ The decrease or disappearance of IKA in the control group may be due to the degradation of IKA channels under long‐term in vitro culture or the decrease in synaptic function.^[^ ^26^ ^]^ The high electrical conductivity of SA‐CNT helped the maintenance of channel functions, which have also been reported in cardiac cells.^[^ ^27^ ^]^ In human cocultured neural organoids, the electrophysiological features of the differentiated SGNs demonstrate the establishment of functional synapses between differentiated peripheral neurons and central neurons in the SA‐CNT system. In particular, the emergence of EPSCs in SGNs in peripheral organoids supports the synaptic connection between neurons.^[^ ^28^ ^]^ Moreover, we implemented a series of neuroscience tools, demonstrating that this system can be used for tracing connectivity, as well as for probing and manipulating neuronal connection. Employing a combination of optogenetic stimulation with calcium imaging and patch clamp recording,^[^ ^29^ ^]^ we observed synchronous activation of neurons in different units in the established human auditory neural circuit. Further work on optimizing the transparency of scaffolds is also necessary to conduct other technologies, such as recording optically evoked calcium responses.

The main advantage of our approach is the construction of neural connections from primary human sources. In contrast to the vast majority of neural organoids induced from human pluripotent stem cells (hPSCs),^[^ ^30^ ^]^ the genomic and behavioral characteristics of primary cells are more similar to those in vivo. Due to the long induction periods and variable differentiation direction, it is relatively difficult to precisely induce region‐specific neurons from hPSCs. Target cells induced from primary progenitor cells preserve the epigenetic and genetic characteristics of the tissue from which they originated, while the availability and scalability of primary progenitor cells are limited, as they rely on donor tissue and may present ethical concerns. In contrast, hPSCs can be induced from various cell types and expanded indefinitely.^[^ ^31^ ^]^ The neurons in organoids can be efficiently differentiated from primary neural progenitor cells, which would be closer to the features of auditory region‐specific neurons, yet this needs to be confirmed by the comparison of genomic sequencing and electrophysiological detection.^[^ ^32^ ^]^ Utilizing the coculture technique, a novel human auditory circuit model was constructed. Notably, the auditory circuits we established bypass the cochlear nucleus, lateral lemniscus complex, and other neural nuclei by directly establishing a connection between the peripheral SGNs and the highest central auditory neurons. However, the role of these auditory nuclei in potential transmission and encoding cannot be ignored,^[^ ^33^ ^]^ and integrating all levels of auditory units can perfectly recapitulate the function of the auditory pathway in future works. The success of establishing direct connections between the peripheral and central nervous systems also provides us with broad insights into the location of auditory implants and strategies for restoring hearing function.

## 5 Conclusion

In summary, we have developed a method to construct human neural peripheral‐central pathways of the auditory system that retain electrically active auditory neurons and functional synaptic connections. The SA‐CNT system has unique advantages in terms of establishing connections of region‐specific neural units and accelerating the search for therapeutics for neuropsychiatric diseases. As a novel auditory circuit model from primary human sources, the system provides a valuable platform to study the physiological interactions of auditory neurons, as well as the developmental features of the human auditory system. We envision that the new model system will be beneficial for exploring effective genetic and pharmacological strategies that re‐establish functional connections between central and peripheral auditory nerves to treat sensorineural hearing loss.

## Conflict of Interest

The authors declare no conflict of interest.

## Supporting information

Supporting Information

## Data Availability

Data sharing is not applicable to this article as no new data were created or analyzed in this study.
