# Correction to [Integration of Functional Human Auditory Neural Circuits Based on a 3D Carbon Nanotube System]

**DOI:** 10.1002/advs.202410897

**Published:** 2024-10-16

Y. Lou, J. Ma, Y. Hu, X. Yao, Y. Liu, M. Wu, G. Jia, Y. Chen, R. Chai, M. Xia, W. Li, Integration of Functional Human Auditory Neural Circuits Based on a 3D Carbon Nanotube System. *Adv. Sci*. **2024**, *11*, 2309617.

https://doi.org/10.1002/advs.202309617

We discovered the duplication of images in Figure 2. The incorrect bright‐filed panels in Figure 2b and Figure 2d were highlighted in the red box. Due to an error in the arrangement of Figure 2d, we pasted the wrong bright‐field images that do not correspond to the fluorescent images. It was not an act of academic misconduct such as falsification. These corrections do not impact the overall findings and conclusions of the paper.


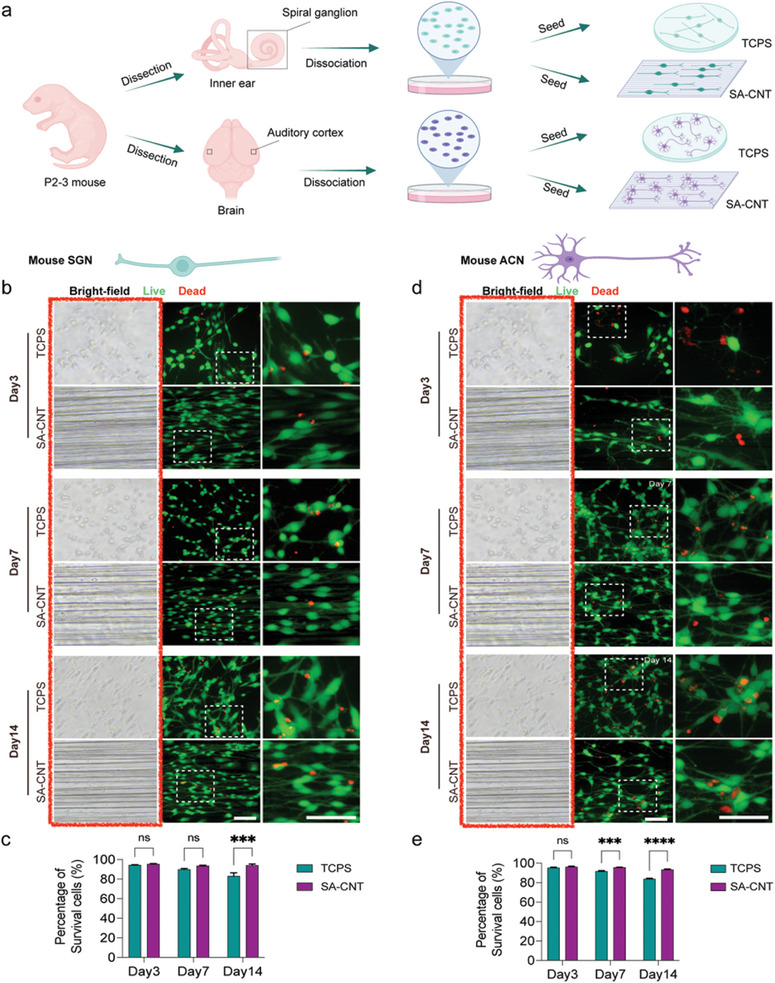


Below is the original version of Figure 2, we corrected the Figure 2d.


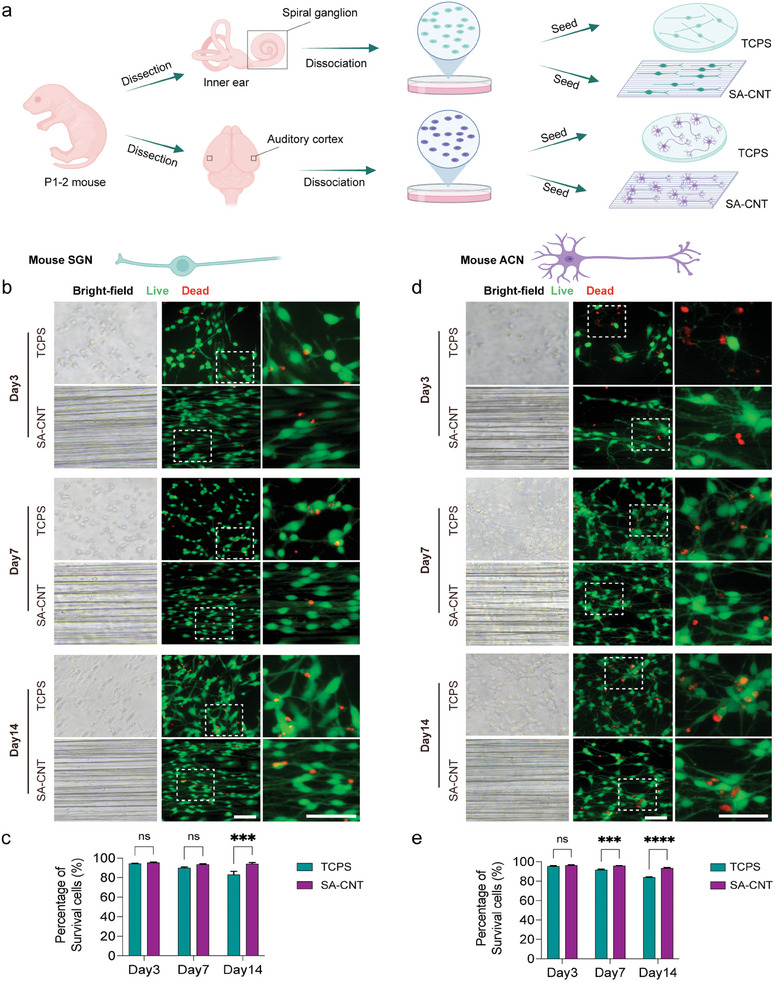


We apologize for this error.

