# Supplementary material for: Integration of Functional Human Auditory Neural Circuits Based on a 3D Carbon Nanotube System
Source: Adv Sci (Weinh). 2024 Jun 18;11(32):2309617. doi: 10.1002/advs.202309617 (PMC11348147; doi:10.1002/advs.202309617)
Supplement: Supplementary file 1 — Supporting Information [file ADVS-11-2309617-s001.docx]

Supporting Information

# Integration of Functional Human Auditory Neural Circuits Based on a Three-dimensional Carbon Nanotube System

*Yiyun Lou^#^, Jiaoyao Ma^#^, Yangnan Hu^#^, Xiaoying Yao^#^, Yaoqian Liu, Mingxuan Wu, Gaogan Jia, Yan Chen, Renjie Chai^*^, Mingyu Xia^*^ and Wenyan Li^*^*


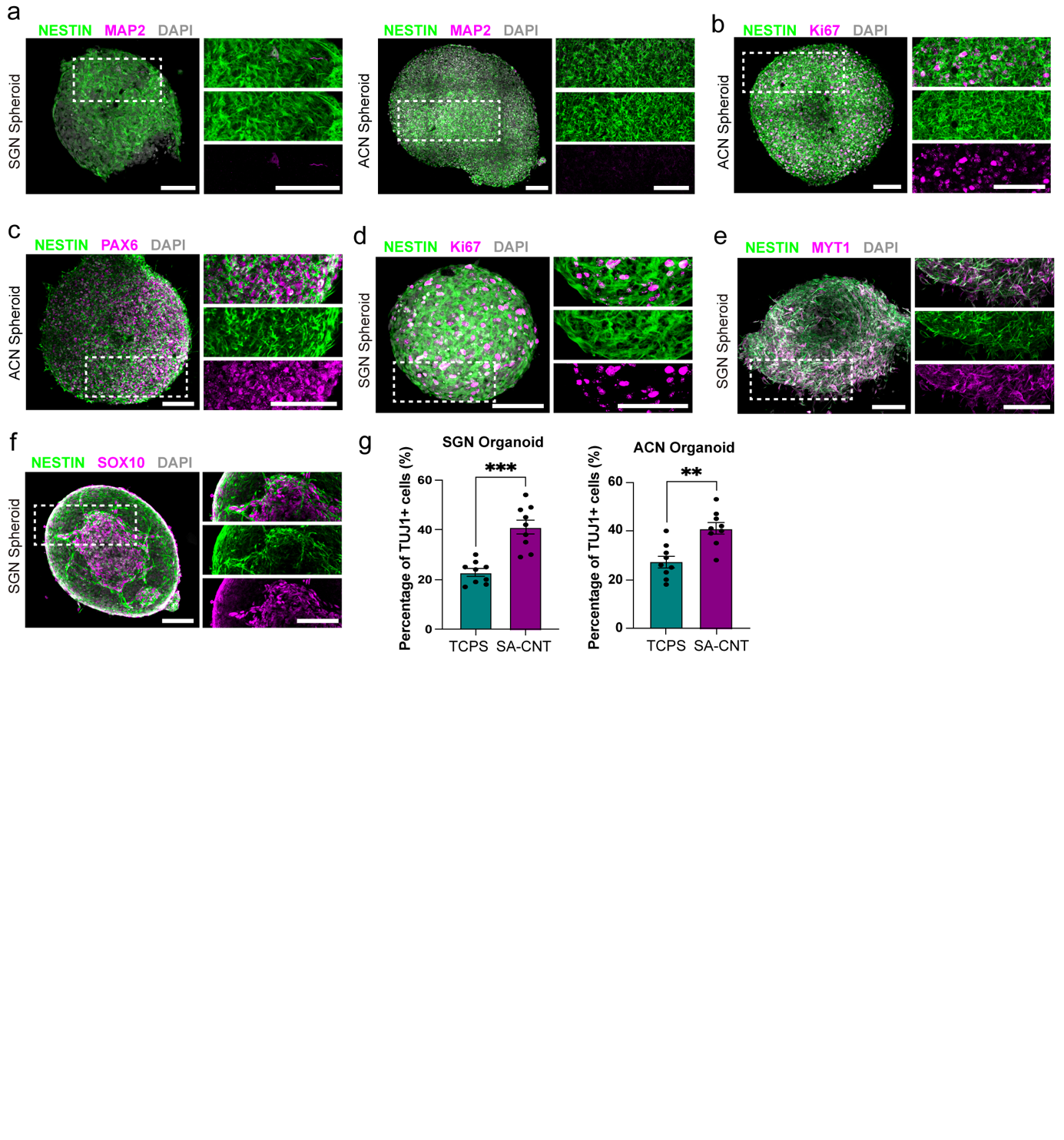


**Figure S1.** (a) Representative confocal images showing the expression of NESTIN and MAP2 in SGN and ACN spheroids on Day 7. (b) Representative confocal images showing the expression of NESTIN and Ki67 in ACN spheroids on Day 7. (c) Representative confocal images showing the expression of NESTIN and PAX6 in ACN spheroids on Day 7. (d) Representative confocal images showing the expression of NESTIN and Ki67 in SGN spheroids on Day 7. (e) Representative confocal images showing the expression of NESTIN and MYT1 in SGN spheroids on Day 7. (f) Representative confocal images showing the expression of NESTIN and SOX10 in SGN spheroids on Day 7. (g) Quantifying the percentage of TUJ1+ cells per organoid. 9 organoids at each condition. The data are presented as the mean ± SEM, **P <0.01, ***P <0.001 (Mann Whitney test).


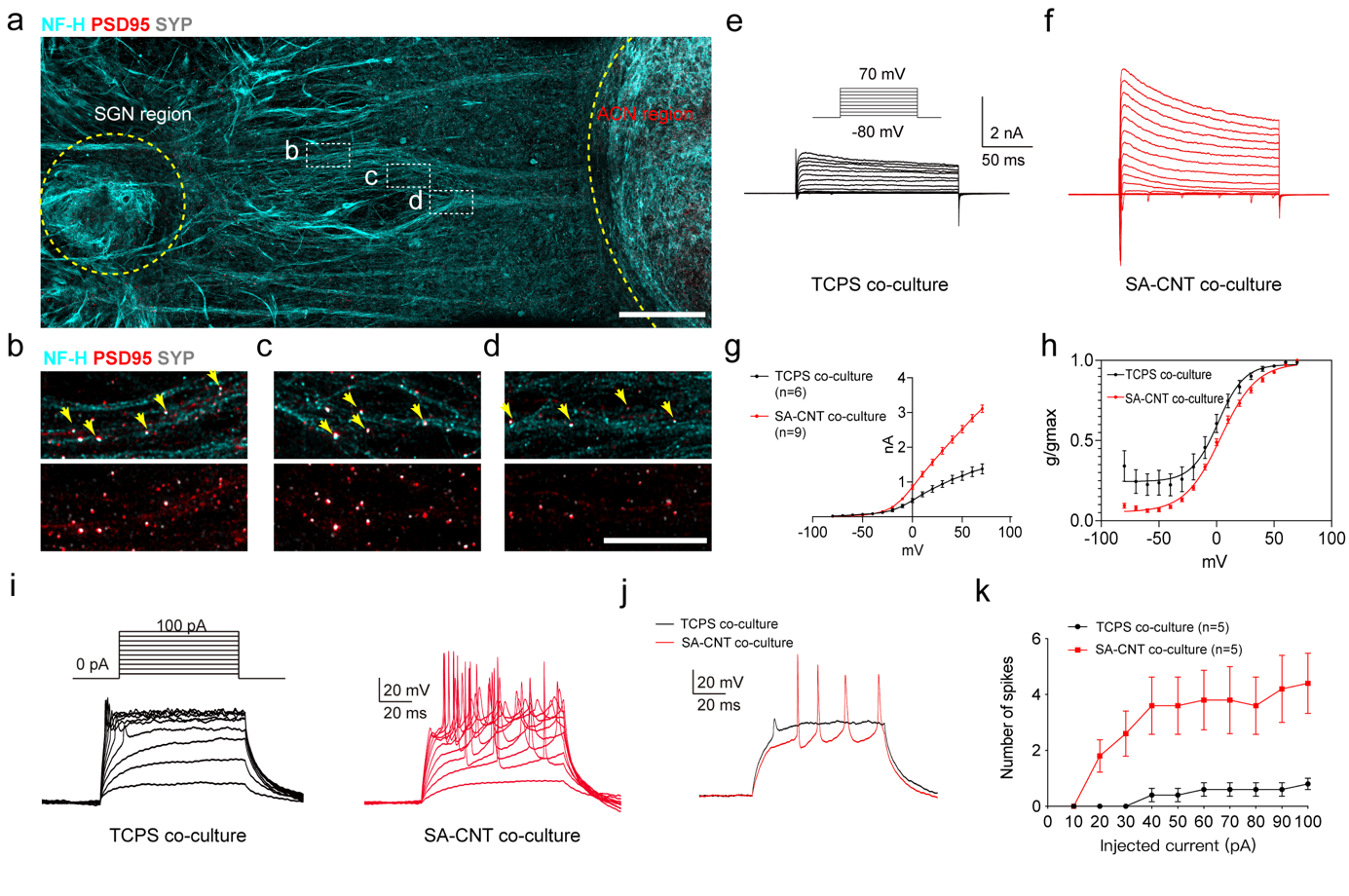


**Figure S2.** (a) Representative confocal images show NF-H, PSD95 and SYP staining of the human SGN and ACN spheroids co-cultured on SA-CNT on Day 20. Scale bar, 200 μm. (b-d) Higher magnification of the selected region showing the SYP+ synaptic vesicles co-localized with PSD95+ postsynaptic vesicles. Scale bar, 20 μm. (e-f) Family of outward potassium currents recorded from two SGNs cultured on TCPS (e) and SA-CNT (f), evoked by the series of voltage steps shown above. (g) Mean ± SEM peak current-voltage relations for 6 SGNs cultured on TCPS (black) and 9 SGNs cultured on SA-CNT (red). (h) Normalized GV relations for outward potassium currents recorded from SGNs cultured on TCPS (black) and SA-CNT (red). Error bars show SEM, and lines show single Boltzmann distribution fits. (i) Representative voltage responses evoked by step current injection of 10~100 pA shown above, recorded from two SGNs cultured on TCPS (black) and SA-CNT (red). (j) Representative voltage responses evoked by 40pA current injection, recorded from two SGNs cultured on TCPS (black) and SA-CNT (red). (k) Number of spikes evoked by step current of 10 ~ 100 pA.
